# Human Apolipoprotein E Resequencing by Proteomic Analysis and Its Application to Serotyping

**DOI:** 10.1371/journal.pone.0085356

**Published:** 2014-01-14

**Authors:** Motoi Nishimura, Mamoru Satoh, Satomi Nishimura, Shoko Kakinuma, Kenichi Sato, Setsu Sawai, Sachio Tsuchida, Takeshi Kazama, Kazuyuki Matsushita, Sayaka Kado, Yoshio Kodera, Fumio Nomura

**Affiliations:** 1 Department of Molecular Diagnosis, Graduate School of Medicine, Chiba University, Chiba, Japan; 2 Division of Laboratory Medicine and Clinical Genetics, Chiba University Hospital, Chiba, Japan; 3 Clinical Proteomics Research Center, Chiba University Hospital, Chiba, Japan; 4 Chemical Analysis Center, Chiba University, Chiba, Japan; 5 Laboratory of Biomolecular Dynamics, Department of Physics, School of Science, Kitasato University, Sagamihara, Japan; National Cancer Center, Japan

## Abstract

**Background:**

Apolipoprotein E (ApoE) typing is considered important because of the association between ApoE and Alzheimer’s disease and familial dyslipidemia and is currently performed by genetic testing (*APOE* genotyping). ApoE levels in plasma and serum are clinically determined by immunoassay.

**Methods:**

Combining an ApoE immunoassay reagent with proteomic analysis using an Orbitrap mass spectrometer, we attempted to resequence ApoE from trace amounts of serum for typing (serotyping). Most (24 of 33) ApoE mutant proteins registered to date with Online Mendelian Inheritance in Man, such as ApoE2 and ApoE4, involve lysine and arginine mutations. Digestion of mutant ApoE with trypsin will thus result in fragments that differ substantially from wild-type ApoE3 in terms of mass, making serotyping ideally suited to mass spectrometry analysis.

**Results:**

The mean coverage of the amino acid sequence of full-length ApoE was 91.6% in the protein resequence. Residues 112 and 158 (which are mutated in ApoE2 and ApoE4) were covered in all samples, and the protein sequences were used for serotyping. Serotypes including all heterozygous combinations (ApoE2/E3, E2/E4, E3/E4) corresponded exactly to the *APOE* genotyping results in each of the subjects.

**Conclusion:**

Our novel ApoE serotyping method with protein resequencing requires no synthesis of stable isotope-labeled peptides or genome analysis. The method can use residual blood from samples collected for routine clinical tests, thus enabling retrospective studies with preserved body fluids. The test could be applied to samples from subjects whose DNA is unavailable. In future studies, we hope to demonstrate the capability of our method to detect rare ApoE mutations.

## Introduction

Humans have three major apolipoprotein E (ApoE) alleles (*APOE*; ε2, ε3, and ε4) that produce three ApoE protein isoforms. The ε2 allele encodes ApoE2 (Cys112, Cys158), whereas ε3 encodes wild-type ApoE3 (Cys112, Arg158), and ε4 encodes ApoE4 (Arg112, Arg158). Because the type of ApoE expressed is believed to be an indicator of dementia risk and familial hyperlipemia, many clinical studies have begun to utilize ApoE typing in recent years [1]. In addition to these major ApoE isoforms, many other ApoE isoforms, such as ApoE-Sendai (Arg145Pro), have been registered to date with Online Mendelian Inheritance in Man.

At present, ApoE type is usually determined through genetic analysis, which requires preservation of DNA. On the other hand, because the ApoE4 isoform involves a single amino acid mutation, enzyme-linked immunosorbent assay (ELISA) kits that use an anti-ApoE4 antibody [2] have been developed. These kits are commercially available for ApoE4 typing directly from tissue (including serum, plasma, cerebrospinal fluid, or brain), and thus are applicable to instances where DNA is not available. This assay has been employed in studies using preserved blood samples and brain tissues [3]–[6]; however, it permits determination only of the presence or absence of ApoE4 and not that of other ApoE types. Furthermore, discrimination of the ApoE4 homozygous combination from its heterozygous combination requires construction of a standard curve.

In recent years, a few studies based on proteomic analyses using mass spectrometry (MS) have attempted ApoE isoform typing as well as quantitative determination of ApoE. A recent report described the use of stable isotope labeling tandem MS for isoform-specific relative quantitation of ApoE4 [7]. This approach is based on comparative measurements of cells labeled in culture with amino acids incorporating stable isotopes. Another study attempted MS-based quantification of ApoE isoforms by labeling full-length ApoE with a stable isotope [8], but this method still requires ApoE genotype information.

Mass spectrometry has progressed tremendously in the past few years, such that it is now possible to resequence a target protein by comparing its mass spectra with data contained in protein databases. ApoE typing is ideally suited to proteomic analyses thanks to these recent advances in MS technology. The initial step of a conventional proteomic analysis involves digesting a target protein with trypsin in order to fractionate it into peptides. Because trypsin digestion cleaves the peptide bond between the carboxyl group of Arg or Lys residues and the amino group of the adjacent residue, ApoE isoforms containing mutations involving Arg or Lys residues (ApoE2 and ApoE4) will produce fragments of differing masses compared with isoforms involving substitution of a single amino acid other than Arg or Lys. Isoforms with mutations involving Lys or Arg should thus be clearly identifiable using MS.

Quantitation of the level of ApoE in blood is typically determined using a commercially available immunoassay. When used as a reagent for immunoprecipitation, the antibodies used to capture ApoE in the immunoassay permit relatively easy isolation of ApoE from serum. Thus, in this study we attempted to type ApoE in serum by combining reagents from a commercially available immunoassay with MS-based proteomic analyses. We achieved resequencing of nearly the full length of the ApoE protein in serum and successfully used the sequence for ApoE serotyping in human subjects.

## Materials and Methods

### Subjects and Serum Samples

Blood samples were collected from 100 apparently healthy Japanese subjects. Written informed consent was obtained prior to sample collection, and the study was approved by the Human Ethics Committee of Chiba University.

ApoE genotyping was performed by direct sequencing of DNA from all subjects using a 3130 Genetic Analyzer (ABI/Life Technologies) and BigDye Terminator v.3.1 cycle-sequence reaction reagent. To cover a wide range of genotypes with minimal bias, 16 samples were selected and the analysts were blinded to genotype.

### Immunoprecipitation

Immunoprecipitation with a commercially available turbidimetric immunoassay kit (N-assay TIA Apo E, Nittobo, Tokyo, Japan) was performed for approximately 15 min. Specifically, this kit comprises two (R-1 buffer and R-2 antibody solution) reagents, and 5 µL of each serum sample was mixed with 12 µL of R-1 reagent and heated in a 37°C water basin for 5 min. Next, 12 µL of R-2 reagent was added and the samples were heated for an additional 5 min at 37°C, after which the samples were centrifuged at 12,400×*g* for 5 min. The supernatant was discarded and the pellet was washed 3 times with 500 µL of phosphate-buffered saline (PBS). The PBS was removed and the proteins in the pellets were carbamidomethylated. Next, 8 µL of sample buffer was added to each sample and the proteins were resolved by sodium dodecyl sulfate-polyacrylamide gel electrophoresis (SDS-PAGE) [9] on a 10–20% continuous gradient gel.

### In-gel Digestion and Protein Identification

Immunoprecipitated proteins were reductively alkylated before SDS-PAGE, after which 1 µL of 200 mM dithiothreitol was added and the mixture was incubated at 57°C for 30 min. Next, 1 µL of 600 mM iodoacetamide was added and samples were incubated at room temperature for 30 min in the dark. Reductively alkylated proteins were then separated by one-dimensional SDS-PAGE. Bands of approximately 34 kDa corresponding to ApoE were visualized by staining the gel with Coomassie brilliant blue. The ApoE bands were individually excised and identified by in-gel tryptic digestion followed by liquid chromatography-tandem mass spectrometry (LC-MS/MS) [10]. The digested peptides (presumed to be derived from ApoE and to be free of antibody contamination) were desalted and enriched using C18-StageTips [11]. One-tenth of each enriched sample was injected onto a C18 trap column (DIONEX, Sunnyvale, CA, USA) in line with a C18 analytical column (Nikkyo Technos, Tokyo, Japan) connected to an Ultimate 3000 LC system (DIONEX). The flow rate of the mobile phase was 300 nL/min. The solvent composition of the mobile phase was programmed to change in 120-min cycles with the following varying mixed ratios: solvent A (2% [v/v] CH_3_CN and 0.1% [v/v] HCOOH) to solvent B (90% [v/v] CH_3_CN and 0.1% [v/v] HCOOH) 5–10% B (5 min), 10–13.5% B (35 min), 13.5–35% B (65 min), 35–90% B (4 min), 90% B (0.5 min), 90–5% B (0.5 min), and 5% B (10 min). Purified peptides eluting from the LC column were introduced into an LTQ-Orbitrap XL (Thermo Scientific, San Jose, CA, USA) hybrid ion-trap Fourier transform mass spectrometer. The Proteome Discoverer search engine (version 1.2, Thermo Scientific) was used to identify proteins from peptide mass spectra and tandem mass spectra. Peptide mass data were matched against peptides contained in the modified UniProtKB human database (SwissProt 2010, November 2010, 20,331 entries) with added amino acid sequences for ApoE2 and ApoE4. The database search parameters were as follows: peptide mass tolerance, 2 ppm; fragment tolerance, 0.6 Da; the enzyme was set to trypsin, allowing up to one missed cleavage; fixed modification, cysteine carbamidomethylation; variable modification, methionine oxidation. The minimum criteria for protein identification were filtered with Xcorr vs. charge state and a false discovery rate (FDR) of <1%. The FDR was estimated by searching against a randomized decoy database created with Proteome Discoverer 1.2 (Thermo Scientific).

### ApoE Serotyping

ApoE isoforms were typed according to the identification results returned by the Proteome Discoverer search engine. The following relationships exist between ApoE genotypes and the isoforms found in the blood. For genotype ε2/ε2, ApoE is present only as the E2 isoform; for ε3/ε3, only the E3 isoform is present in the blood; for ε4/ε4, only the E4 isoform is present. For genotype ε2/ε3, ApoE is present in the blood as E2/E3; for ε2/ε4, ApoE is present as E2/E4; for ε3/ε4, ApoE is present as E3/E4. Because of the correlation between ApoE genotype and isoform, it is possible to determine the genotype from the blood ApoE isoform combination.

### Statistical Methods

Numerical data are presented as the mean ± SD (standard deviation). The results were analyzed using the 4-Step Excel Statistics software application (OMS Publishing Inc., Tokorozawa, Japan, http://www.oms-publ.co.jp/index.html).

## Results

### ApoE Amino Acid Sequence Determination by LC-MS/MS

**Figure 1:**
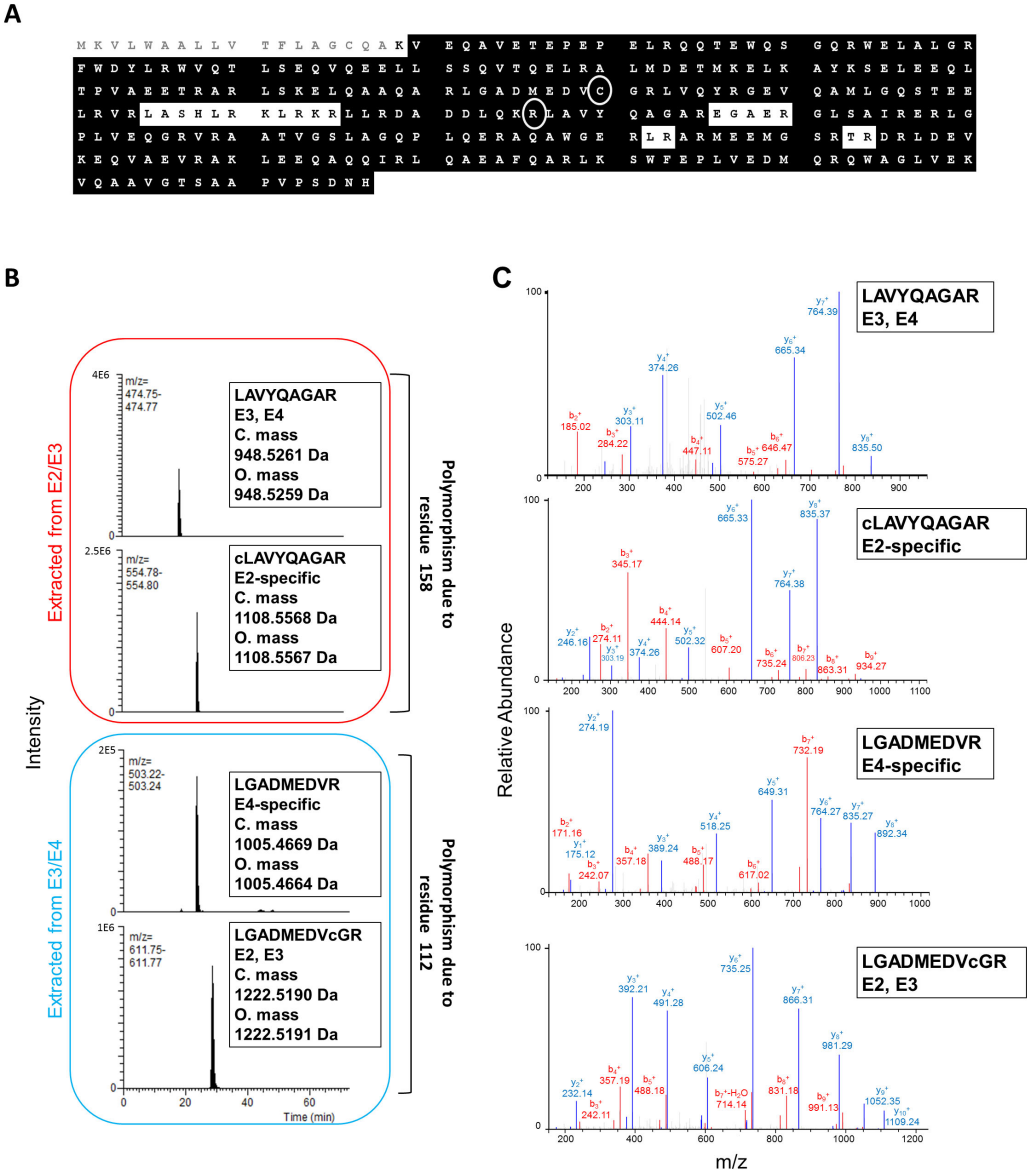
Apolipoprotein E resequencing and its application to serotyping. (**A**) ApoE resequencing. Figure shows a representative result of wild-type ApoE amino acid sequence determination (sequence coverage = 93.6%, excluding the 18-residue signal peptide) using Orbitrap LC-MS/MS. Black highlighting denotes the determined sequence. Amino acid residues C112 and R158, which demonstrate polymorphism in ApoE2 (C158) and ApoE4 (R112), are circled. Amino acids are represented by their one-letter codes. (**B**) Tryptic peptide polymorphisms and ion chromatograms. Mutations in amino acid residues 112 and 158, which were covered by protein resequencing, cause peptide fragment polymorphisms. The R158C mutation (ApoE2) results in the cLAVYQAGAR peptide, where the C112R mutation (ApoE4) yields the LGADMEDVR peptide. Figure shows representative chromatograms for the doubly charged ions extracted from subjects with E2/E3 and E3/E4 heterozygous combinations. The calculated and observed monoisotopic masses for each peptide are indicated. (**C**) Corresponding MS/MS spectra for each peptide in (**B**). Polymorphic peptide sequences from subjects with heterozygous combinations were confirmed by MS/MS. The b- and y-ions are labeled. In (**B**) and (**C**), lower-case “c” represents alkylated cysteine residues. C. mass = calculated mass; O. mass = observed mass; Da = dalton.

Using the serum samples obtained from the 16 subjects enrolled in the study, we determined the primary amino acid sequence of the ApoE isoforms contained in each sample using LC-MS/MS on an Orbitrap mass spectrometer. The mean sequence coverage for full-length ApoE was 91.6±4.57% (SD), excluding the 18-residue signal peptide (Figure 1, Table 1). An example illustrating 93.6% sequence coverage is shown in Figure 1A. The LC-MS/MS analysis with approximately 90% sequence coverage in this study amounts to protein resequencing of nearly the full length of ApoE, which may be useful for making unequivocal determinations of several ApoE isoforms (Figure 1A). Indeed, the peptides containing amino acid residues 112 and 158, which are mutated in ApoE2 and ApoE4, were also covered by the protein resequencing in all 16 subject samples. Tryptic peptide polymorphisms due to the mutated amino acid residues 112 and 158 were detected with abundant signal in each single LC–MS run (Figure 1B). The matching b- and y-ions for each peptide were identified in their respective mass spectra (Figure 1C).

**Table 1:** Comparison of *APOE* genotypes with results of APOE protein resequencing and serotyping.

Genotype	n	Mean percent sequence coverage (±SD)	Serotyping result
**ε2/ε3**	2	87.0 (N.D.)	E2/E3
**ε2/ε4**	1	93.6 (N.D.)	E2/E4
**ε3/ε4**	6	92.9 (±1.85)	E3/E4
**ε4/ε4**	2	90.5 (N.D.)	E4/E4
**ε3/ε3**	5	91.8 (±5.78)	E3/E3
**Total**	16	91.6 (±4.57)	*

The serotypes agreed with the genotypes for all enrolled subjects.

n = number of subject; SD = standard deviation; N.D. = not determined.

### ApoE Serotyping

**Figure 2:**
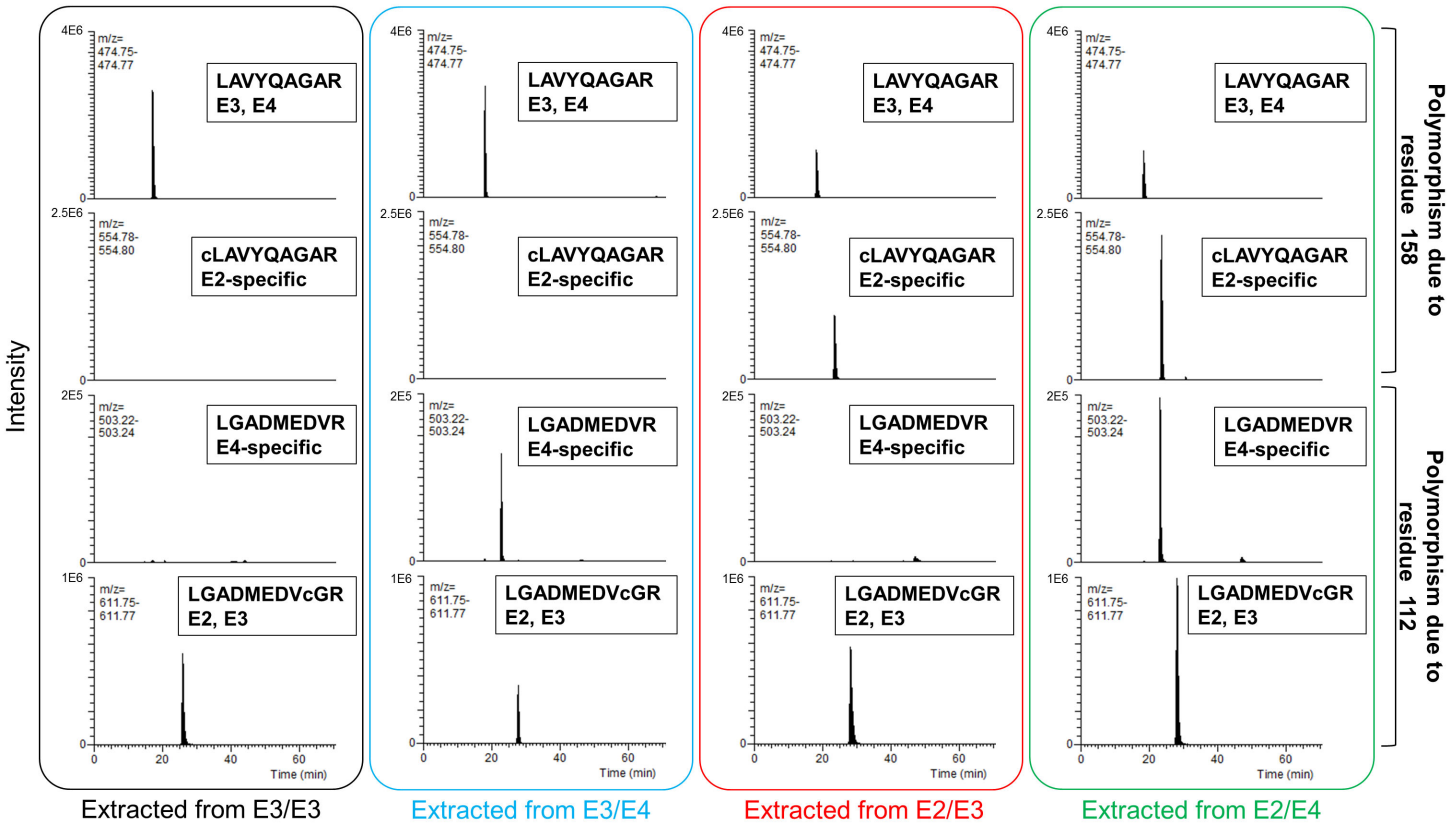
Serotyping in heterozygous combinations. Figure shows representative ion chromatograms of the wild-type (E3/E3) and all heterozygous combinations (E2/E3, E3/E4, E2/E4). Tryptic peptide polymorphisms correspond to each ApoE isoform. As described under “ApoE serotyping” in the **Materials and Methods**, the correlation between ApoE genotypes and isoforms enables determination of the genotype from the blood ApoE isoform combination.

Having confirmed that the polymorphic amino acids were covered in the proteomics-based sequence analyses, we then applied the sequence to ApoE serotyping. All analysts were blinded with respect to the subjects’ genotypes and typed the ApoE isoforms based upon the identification results returned by the Proteome Discoverer search engine. The serotypes agreed with the genotypes for all 16 subjects (Table 1). Tryptic peptide polymorphism detection in representative cases (a wild-type E3/E3 and all heterozygous combinations (E2/E3, E3/E4, E2/E4)) are shown in Figure 2.

## Discussion

We demonstrate here a method for serotyping ApoE that involves immunoprecipitation from a relatively small amount of serum sample (5 µL) followed by protein resequencing using LC-MS/MS with an Orbitrap mass spectrometer. The method does not require genome analysis but provides highly accurate genotype information (Table 1). Commercially available clinical immunoassay reagents are used for immunoprecipitation, and the ApoE serotyping does not require synthesis of stable isotope-labeled peptides and proteins.

In a previous study, in which ApoE polymorphism was detected using mass spectrometry and stable isotope-labeled protein, most isoforms could be quantified, but not all isoform types could be detected without genotyping results in the case of heterozygous combinations [8], while our method allows for the detection of all heterozygous combinations (ApoE2/E3, E2/E4, and E3/E4) without genotyping (Table 1, Figure 2).

In the field of hemoglobinopathy, amino acid sequence variations have been identified using a MS-based proteomic method without hemoglobin gene information [12]. In that study, the authors performed isoelectric focusing on agarose plates, and hemoglobin proteins were isolated by cutting from the gel, followed by proteomic analysis with high-performance liquid chromatography/microspray tandem mass spectrometry. Hemoglobin is an abundant protein present in blood, and thus an isoelectric focusing method could be applied; ApoE is a less abundant protein and our method employed immunoprecipitation with a commercial clinical immunoassay reagent for ApoE isolation.

In addition to the ApoE2, E3, and E4 isoforms, many other ApoE isoforms, such as ApoE-Sendai (Arg145Pro), have been reported, despite being present at low frequencies in the population [13]. As of July 2013, 33 ApoE mutant alleles were registered with Online Mendelian Inheritance in Man. A total of 24 mutations in these alleles are associated with Lys and Arg residues, including mutations in ApoE2 and ApoE4. Isoforms carrying these mutations will produce tryptic digestion fragments that differ substantially in mass compared with fragments from an isoform with a simple substitution of a single amino acid other than Lys or Arg. Thus, the MS-based extensive resequencing (Figure 1A) and serotyping method should enable unambiguous identification of corresponding isoforms. However, because recent advances in MS technology have made the technique sophisticated enough to enable detection of a single amino acid substitution, in theory our method should be capable of identifying mutations that are not associated with Lys or Arg residues. In addition, our method does not require synthesis of control peptides for each target isoform; thus, we expect that it would enable detection of not only ApoE2/E3/E4, but also many other ApoE isoforms. It is likely that there is some pathophysiological significance to the association of ApoE mutations with Lys or Arg residues; our method may help researchers gain insight into this matter, which may be difficult to elucidate through genotyping alone.

In the diagnosis of dyslipidemia and risk assessment of dementia, ApoE typing is important. Our method enables ApoE serotyping using a small amount of serum, and as such the method may be useful for ApoE analysis of blood remaining from samples collected for routine clinical tests. Moreover, our method may enable retrospective studies using preserved body fluids or serum samples from anonymous subjects for whom DNA is not available. In future studies, we hope to demonstrate the capability of this resequencing and serotyping method to detect rare ApoE mutations.
